# Contrast-Based 3D/2D Registration of the Left Atrium: Fast versus Consistent

**DOI:** 10.1155/2016/7690391

**Published:** 2016-03-08

**Authors:** Matthias Hoffmann, Christopher Kowalewski, Andreas Maier, Klaus Kurzidim, Norbert Strobel, Joachim Hornegger

**Affiliations:** ^1^Pattern Recognition Lab, Friedrich-Alexander-University Erlangen-Nuremberg, 91058 Erlangen, Germany; ^2^Cardiac Arrhythmia Service, Hospital Barmherzige Brueder, 93049 Regensburg, Germany; ^3^Erlangen Graduate School in Advanced Optical Technologies (SAOT), 91052 Erlangen, Germany; ^4^Siemens Healthcare GmbH, 91301 Forchheim, Germany

## Abstract

For augmented fluoroscopy during cardiac ablation, a preoperatively acquired 3D model of a patient's left atrium (LA) can be registered to X-ray images recorded during a contrast agent (CA) injection. An automatic registration method that works also for small amounts of CA is desired. We propose two similarity measures: The first focuses on edges of the patient anatomy. The second computes a contrast agent distribution estimate (CADE) inside the 3D model and rates its consistency with the CA as seen in biplane fluoroscopic images. Moreover, temporal filtering on the obtained registration results of a sequence is applied using a Markov chain framework. Evaluation was performed on 11 well-contrasted clinical angiographic sequences and 10 additional sequences with less CA. For well-contrasted sequences, the error for all 73 frames was 7.9 ± 6.3 mm and it dropped to 4.6 ± 4.0 mm when registering to an automatically selected, well enhanced frame in each sequence. Temporal filtering reduced the error for all frames from 7.9 ± 6.3 mm to 5.7 ± 4.6 mm. The error was typically higher if less CA was used. A combination of both similarity measures outperforms a previously proposed similarity measure. The mean accuracy for well contrasted sequences is in the range of other proposed manual registration methods.

## 1. Introduction

**Figure 1:**
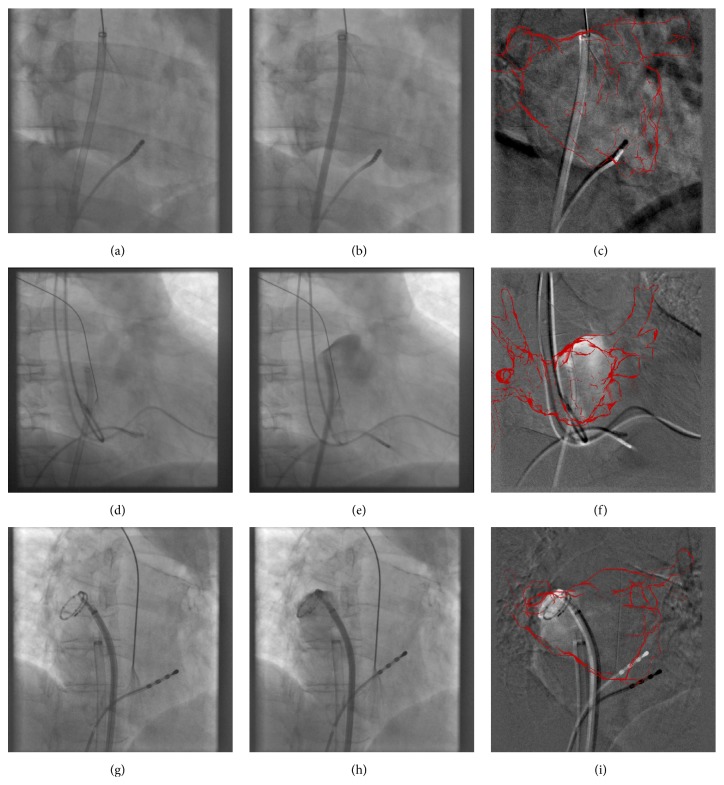
Three different contrast agent injections. The left column shows an uncontrasted frame, the middle column shows a contrasted frame of the sequence, and the rightmost column shows a subtraction of both images. The manually established ground-truth position of the left atrium is overlaid with red. The LA in image (c) appears only faintly contrasted and the CA just flows into the ventricle. On the other hand, image (f) shows disturbing motion artifacts caused by leads and wires associated with an implanted device. In image (i), the contrast agent is injected into a pulmonary vein and catheter motion artifacts are visible. While the spine in the center of this image vanishes, soft-tissue motion artifacts related to the lungs are visible at the left and right border, respectively.

Atrial fibrillation is the most common heart arrhythmia affecting around 2.2 million people in the USA. A possible treatment option is catheter ablation, which is a minimally invasive procedure. Depending on the ablation device used, it is carried out using either electroanatomic mapping systems, an X-ray guided approach, or a combination of both. X-ray guidance is, for example, required if a cryoballoon is used for ablation as current cryoballoons cannot be directly located by electroanatomic mapping systems. In this paper, we focus on X-ray guided approaches. One weakness of X-ray imaging is poor soft-tissue contrast. As a consequence, the left atrium (LA) can only be seen if contrast agent (CA) is injected. However, to reduce the risk of contrast-induced nephropathy, physicians try to keep the use of CA to a minimum often highlighting only a part of the left atrium. To provide orientation to the physician when no CA is present, a model of the patient's LA, for example, generated by a preoperative CT or MRI scan of the patient can be overlaid [1]. The use of such an overlay was found to reduce procedure time and fluoroscopy time significantly [2]. Since a preprocedural 3D scan is often acquired to obtain prior knowledge about a patient's anatomy of the left atrium and to rule out unusual pulmonary vein configurations, it is readily available for augmented fluoroscopy applications. In general, however, the coordinate systems of the preprocedurally acquired 3D heart model and the patient during the intervention differ, and a registration step is usually needed. Today, this registration is usually carried out manually. If the 3D heart model was acquired using CT, several authors proposed to use further landmarks in addition to the LA for registration having obtained associated 3D models by segmentation. Examples are the carina [3], the coronary sinus (CS) [4, 5], the spine [6], or a combination of spine and heart anatomy [7]. For 3D volumes acquired by MRI, for example, MRI angiographies (MRAs), however, a segmentation of the CS or the carina can be very challenging or has not yet been adopted for routine clinical procedures. Fortunately, registration based on contrast injection has been shown to be a fast and accurate alternative [8]. A good moment to obtain contrast-enhanced X-ray images is after the transseptal puncture, in particular if physicians use contrast injections to verify puncture success [9]. Example images of such injections are shown in Figure 1 to illustrate the images available. Since manual registration either needs the attention of the treating physician or requires a trained assistant, more automatic registration methods are desirable.

*(1) Related Work*. There is a significant body of research on registration of 3D objects to 2D fluoroscopic images, for example, for bones [10–12], implants, joints [13], or vessels [14]. An overview is given by Markelj et al. [15]. Compared to implants, a registration of the LA is more complicated for at least two reasons: First, for implants and bones, all parts of the object are visible. During a contrast injection, however, only a part of the left atrium may appear under X-ray.

Second, depending on the amount of CA injected, the overall LA visibility may be poor. For example, in our case EP physicians use contrast at the beginning of the procedure to verify the success of the transseptal puncture. It may also be used later to enhance the anatomy, for example, to make sure that a catheter, for example, a circumferential mapping catheter or a cryoballoon catheter, was placed correctly. This is different to vessel angiography. In this case, higher amounts of contrast are injected to derive diagnostic information, for example, about a stenosis. For ablation procedures in the LA, the CA density and so the visibility of the LA under X-ray may be poorer as the small amount of contrast is injected into the high blood volume of the LA. As a consequence, further effort is needed to develop robust registration methods that can also be applied if CA is used sparingly.

Currently, very few publications deal with registration based on contrast agent: In a first approach towards automatic LA registration, Thivierge-Gaulin et al. [16] tried to find a 3D pose of a model such that its projected shadow matches the contrasted area in a selected image, enhanced by digital subtraction angiography (DSA), best. However, if only a small amount of contrast agent is injected into a somewhat large chamber such as the left atrium, this approach will not lead to a distinct optimum, because the anatomy may not be fully opacified.

Based on CT images, a second approach by Zhao et al. [17] relied on digitally rendered radiographies (DRRs) of the segmented left atrium. The rendered image was compared to a DSA image using normalized gradient correlation. This approach uses a weighting scheme that puts the focus on the roof of the LA. How well this method performs for injections into other areas of the LA is still unclear.

Besides contrast-based registration, 3D data and 2D data can also be aligned using devices. A feature-based method by Sra et al. [4] uses a segmentation of the coronary sinus (CS) in a CT volume to register a 3D LA model to a single 2D fluoroscopic image. A further approach by Brost et al. [5] uses a segmentation of the CS in an MRI volume and a 3D reconstruction of the CS catheter from two fluoroscopic images. Unfortunately, how well the CS can be extracted from a 3D MRI data set depends very much on the MRI scan protocol. Furthermore, due to the strong motion of the CS, it is difficult to relate the position of the CS catheter to the position of the LA, especially for patients having no sinus rhythm but heart arrhythmia [18, 19].

*(2) Contributions and Outline of This Work*. At the beginning of Section 2, we discuss the use of the normalized cross-correlation for registration of a 3D object to 2D X-ray projections based on extracted contrast agent. Afterwards, we propose two new registration techniques for contrast agent-based registration. They require a segmented left atrium as input and can, in contrast to [17], accommodate both CT and MRI data. The first method is described in Section 2.2. Here, we take explicitly apparent edges extracted from a 3D model and compare them to LA edges present in the fluoroscopic images as proposed by [10, 11] for automatic registration of bones and by [20] for manual registration of the LA, respectively. Although our method is conceptually similar to a DRR generation followed by gradient correlation, this calculation can be carried out on a GPU much more quickly than a DRR.

The second contribution described in Section 2.3 is the introduction of a novel similarity measure for biplane X-ray that is tailored to cases in which only parts of an object are visible. Based on a 3D model of the LA, our second method estimates the contrast agent distribution inside the 3D object from a simultaneously acquired pair of fluoroscopic images taken under two different view angles. Then we evaluate how consistent the contrast agent distribution estimate (CADE) is with the acquired fluoroscopic images. As the CADE depends on the transformation used for registration, the transformation leading to the most plausible CADE is used as final position estimate. A brief description of both methods was previously published by us [21].

In Section 2.4 we propose to treat the registration results for each frame of the sequence no longer as independent. Based on our previous publication [22], we use a Markov chain approach to exploit the temporal dependency between successive frames instead.

In Section 3, we evaluate a similarity measure based on the projected shadow which is close to the approach by Thivierge-Gaulin et al. [16] and the two new methods as well as combinations of them. The results are discussed in Section 4.

## 2. Registration Method

For registration, two X-ray sequences showing a CA injection are used. These sequences are acquired simultaneously from two different angles using a biplane system. For each plane, the projection matrix that describes the X-ray camera setup is known. We denote the associated projection operator by *P*_A_ and *P*_B_ for the A-plane and the B-plane of the system, respectively. We also assume that a 3D model of the patient's LA is available, either as a triangle mesh or as a binary volume, as they can be converted into each other.

### 2.1. Contrast Agent Extraction

The contrasted area is found based on a difference image (DSA) **I**_DSA_ = **I**_*u*_ − **I**_*c*_, **I** ∈ *ℝ* ^*m*×*n*^ involving a frame **I**_*c*_ that contains contrast agent and an uncontrasted frame **I**_*u*_. To distinguish between contrasted and uncontrasted frames, either manual annotation or a learning based method can be used, for example, the method described in [23]. Depending on the chosen contrasted frame **I**_*c*_, **I**_DSA_ may contain artifacts, for example, due to motion of the diaphragm or from catheters, if they are at different positions in **I**_*u*_ and **I**_*c*_. Such motion artifacts depend, unlike the information about contrast agent, to a large degree on the choice of **I**_*u*_. For example, if the catheters in **I**_*u*_ are at the same position as in **I**_*c*_, their intensities cancel out. Otherwise, **I**_DSA_ has high positive values at the position of the catheter in **I**_*c*_ and high negative values at the position of the catheter in **I**_*u*_. To keep motion artifacts to a minimum, we propose a best reference selection, which chooses an appropriate reference frame $\hat{I_{u}}$ that matches the chosen contrasted frame **I**_*c*_ as much as possible. Out of all uncontrasted frames, that frame $\hat{I_{u}}$ is selected which minimizes the *L*_1_-norm of the resulting DSA image:

(1) $$\begin{array}{c} \hat{I_{u}}=\underset{I_{u}}{\arg\;\min}\overset{n}{\underset{x=0}{\sum }}\;\overset{m}{\underset{y=0}{\sum }}\left|\;I_{u}\left(x,y\right)-I_{c}\left(x,y\right)\;\right|. \end{array}$$

By following (1), we get frames for which the catheters and the diaphragm cancel out as much as possible. See Figure 3(b) for an example. In **I**_DSA_, only pixels with positive values contain contrast agent. To extract them, we set the intensity of pixels with negative value to 0. Afterwards, we compute a filtered image **I**_*f*_ by applying a median filter with a large kernel size. Smaller structures, for example, caused by motion artifacts that remained despite the optimized choice of **I**_*u*_, do not pass this filter, and the noise in the contrasted area is reduced as well. Finally, a binary image **I**_thr_ of the filtered image **I**_*f*_ is computed using a threshold at *μ*_*f*_ + *σ*_*f*_, where *μ*_*f*_ and *σ*_*f*_  denote the mean and standard deviation of **I**_*f*_, respectively. Thus, a contrasted pixel **p** ∈ *ℝ* ^2^ is indicated by **I**_thr_(**p**) = 1.

A previous approach [16] tried to find a transformation *T* of the 3D model such that the projected shadows **S**_*T*_^A^, **S**_*T*_^B^ of the model into the A-plane and the B-plane of a biplane C-arm system fit best to the contrasted region. Using the normalized cross-correlation (NCC), denoted as *ρ*_*n*_, of two images **I**_1_, **I**_2_ with corresponding mean values *μ*_1_, *μ*_2_ and standard deviations *σ*_1_, *σ*_2_

(2) $$\begin{array}{c} \rho_{n}\left(\;I_{1},I_{2}\;\right)=\overset{n}{\underset{x=0}{\sum }}\;\overset{m}{\underset{y=0}{\sum }}\frac{\left(I_{1}\left(x,y\right)-\mu_{1}\right)\cdot \left(I_{2}\left(x,y\right)-\mu_{2}\right)}{\sigma_{1}\cdot \sigma_{2}}, \end{array}$$

the similarity of the projected shadow and **I**_DSA_ can be measured. Instead of **I**_DSA_, one can also use the binary version of it, that is, **I**_thr_. Then a registration transformation can be estimated by maximizing either one of the two functions

(3) ρshadDSAIDSAA,IDSAB,T=ρnIDSAA,STA·ρnIDSAB,STB,ρshadthrIthrA,IthrB,T=ρnIthrA,STA·ρnIthrB,STB.

### 2.2. Edge Feature

**Figure 2:**
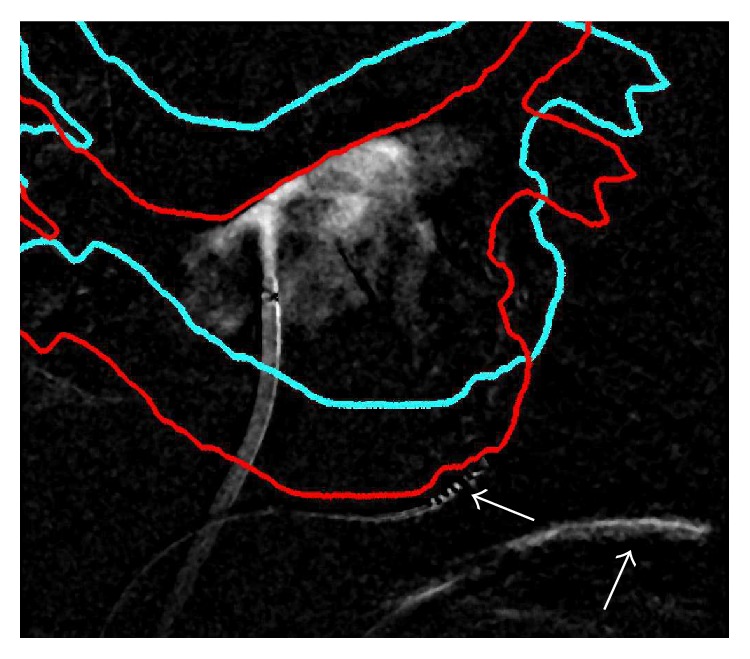
A correct (red) and wrong (cyan) registration result. In both cases, the contrasted area is fully inside the projection shadow represented by the colored outlines. Both registration results lead to a similar NCC value when using an area-based feature for automatic registration. Note that motion artifacts could be kept to a minimum thanks to the best reference frame selection. Only some residual artifacts remained in the vicinity of the moving coronary sinus (CS) catheter and the diaphragm; see white arrows.

**Figure 3:**
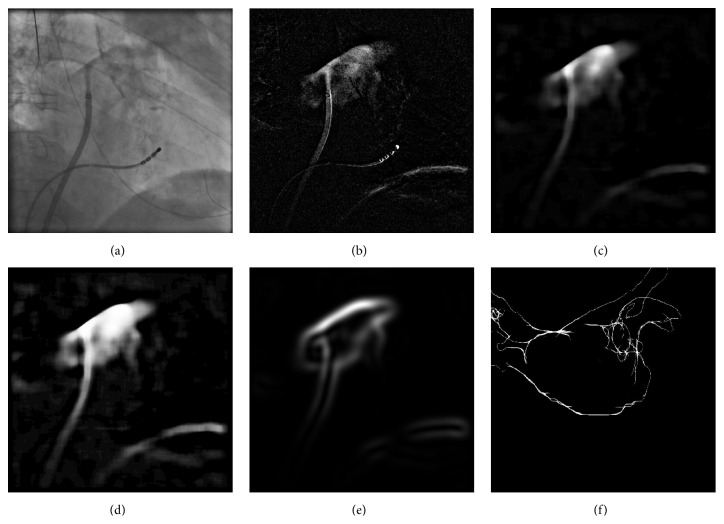
Using the original image (a), a DSA image (b) is computed. After median filtering (c), the remaining motion artifacts from the catheter have vanished. Only the boundary at the diaphragm remained. Afterwards, all pixels are weighted by a sigmoid function (d) to get a more homogeneous value distribution inside the contrasted area. Edges are extracted using derivatives of Gaussian with a large kernel size (e). Finally, the similarity to the rendered edges (f) is evaluated.

Unfortunately, a registration approach only based on contrasted area has multiple solutions if the amount of CA is so little that it does not completely fill the LA; see Figure 2. Fortunately, CA is often injected against the roof or into the pulmonary veins. This results in perceivable edges of the contrasted area which can then be used as registration features. Edge-based registration can be carried out using only the silhouette boundary of the projected object [13] (see Figure 2) or all apparent edges [10, 11] (see Figure 3(f)).

We decided to consider all apparent edges to improve robustness against injections of small amounts of CA, as the silhouette-based approach requires that the LA is contrasted in its entirety. For a partially contrasted left atrium, internal contours may, however, also appear in the fluoroscopic images. This was already found to be beneficial for manual LA registration [20]. Instead of considering edges implicitly by comparing the DSA image to a DRR using gradient correlation [17], we computed them explicitly. To extract edges in the fluoroscopic images, we used the filtered image **I**_*f*_. After applying a median filter, edge-like variations* inside* the contrasted areas may remain. They, however, correspond rarely to anatomical structures and would trigger a response, if an edge filter was applied. To obtain an edge response only at the boundaries of the contrasted area, the image needs to be homogenized before applying an edge filter. Using a simple threshold method would result in a loss of the intensity drop-off at the boundary which provides important information about the edge intensity. Therefore, we weigh all image pixels by a sigmoid function

(4) $$\begin{array}{c} I_{\text{sig}}\left(\;x,y\;\right)=\frac{1}{1+e^{-\left(I_{f}\left(x,y\right)+t\right)\cdot s}}. \end{array}$$

The value of *t* is set to *μ*_*f*_ − *σ*_*f*_, and the parameter *s* depends on the pixel intensity range of the input image. An example of **I**_sig_ is given in Figure 3(d). Finally, **I**_sig_ is filtered using a derivative of Gaussian (DOG) filter to obtain the edge image **I**_DOG_. The kernel size of the DOG filter is set to a large value to get a smooth similarity measure; see Figure 3(e).

The projection of the 3D triangle mesh edges into 2D is done differently than in [10, 20]. We rendered the whole surface mesh and, depending on the viewing direction **d** and the surface normal **n** at a point, we set the opacity *o* of projected triangles to *o* = 1 − |(**d**∘**n**)|; see Figure 3(f) for an example. By doing so, areas that are parallel to the imaging plane are rendered transparent while areas with a normal vector orthogonal to the viewing direction are rendered opaque. The similarity between edges extracted from the fluoroscopic images and the edge images **E**_*T*_^A^, **E**_*T*_^B^ rendered from the 3D model transformed by *T* is measured by

(5) $$\begin{array}{c} \rho_{\text{edge}}\left(\;I_{\text{DOG}}^{A},I_{\text{DOG}}^{B},T\;\right)=\rho_{n}\left(\;I_{\text{DOG}}^{A},E_{T}^{A}\;\right)\cdot \rho_{n}\left(\;I_{\text{DOG}}^{B},E_{T}^{B}\;\right). \end{array}$$

### 2.3. Contrast Agent Distribution Estimation (CADE)

**Figure 4:**
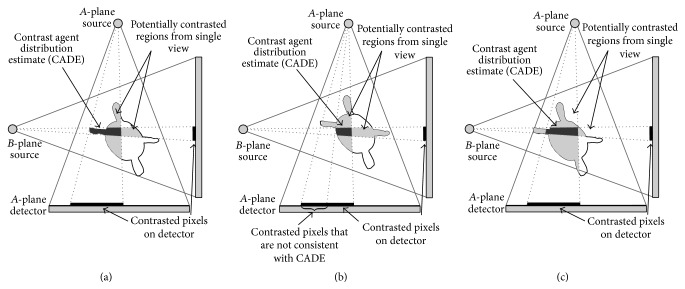
Top-down view on a left atrium and associated contrast-enhanced projections showing a correct registration (a) and two misregistrations (b) and (c). When looking at the contrasted pixels on each detector independently, all LA positions seem to be feasible. Considering the LA position in (b), the area, which can contain contrast agent according to the combination of both detectors, is partially outside the LA. For the CADE, however, only contrast agent inside the LA is considered. So the registration in (b) gives rise to contrasted pixels on the A-plane detector that cannot be explained by the CADE. This will result in a low CADE value and indicate misregistration. There exist, however, also misregistrations that can lead to a consistent CADE as shown in (c). This is why a combination of CADE and edge-based methods yields improved results.

Previous approaches [16, 17] for LA registration searched for a rigid transformation of the LA such that either its projected shadow or its DRR fit to the contrasted area in both fluoroscopic images. 3D information was taken into account insofar as the resulting projections came from the same 3D position of the model. However, such an approach does not necessarily guarantee that corresponding objects in both fluoroscopic images are matched to the same 3D structure of the LA. More precisely, the registration result could be such that, in plane A, the contrast agent is located in a left pulmonary vein (PV) whereas, in plane B, the contrasted area corresponds to a right PV. This is possible as for a given 2D registration in one plane the 2D registration in the other plane has one degree of freedom, which corresponds to an out-of-plane motion in the first plane. An illustration of this problem can be found in Figure 4.

To solve this problem, we compute for a given transformation *T* a CADE inside the LA using binary reconstruction. Then, *T* is optimized such that the contrast agent distribution estimate is most consistent with the projection images. More precisely, a voxel **v** is estimated as contrasted if it satisfies all of the following conditions: (a) the voxel **v** transformed by *T* is projected on a contrasted pixel in plane A, (b) **v** is projected on a contrasted pixel in plane B, and (c) **v** is part of the left atrium (as contrast agent can only be found inside the LA). To compute the CADE, we define the indicator function

(6) $$\begin{array}{c} \chi \left(\;v\;\right)=1⟺v\in R^{3}\text{ is inside the left atrium}. \end{array}$$

Given the binary images **I**_thr_^A^ and **I**_thr_^B^ with corresponding projection operators *P*_A_, *P*_B_ and the indicator function *χ*(**v**), the CADE *C*_*T*_^3D^ for a transformed voxel, *T*(**v**), can be computed as

(7) $$\begin{array}{c} C_{T}^{3D}\left(\;v\;\right)=I_{\text{thr}}^{A}\left(\;P_{A}\left(\;T\left(\;v\;\right)\;\right)\;\right)\cdot I_{\text{thr}}^{B}\left(\;P_{B}\left(\;T\left(\;v\;\right)\;\right)\;\right)\cdot \chi \left(\;v\;\right) \end{array}$$

for a given rigid transformation *T*. This product is the mathematical equivalent of the three conditions introduced above.

**Figure 5:**
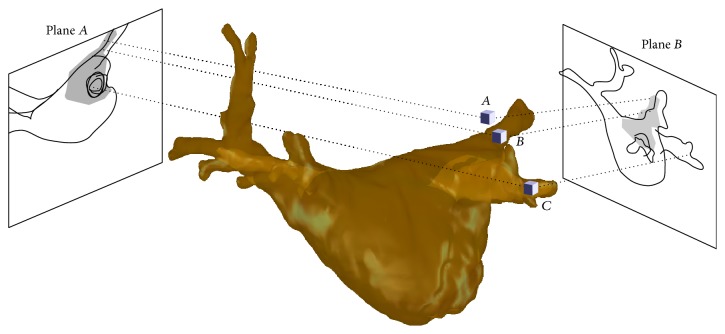
For the transformation shown, only voxel B is estimated as containing CA as it is inside the LA and contrasted in both planes. Voxel C is only contrasted in plane A but not in plane B. Voxel A is filled with contrast in both planes, but it is outside of the LA and therefore considered as uncontrasted. If the LA is moved such that its projections in A and B cover the contrasted area, this voxel will also be considered as contrasted, hence, increasing the consistency with the CADE.

If *T* is chosen suboptimally, the resulting 3D CADE will be inconsistent with the CA observed in the 2D images. That is, a pixel in the 2D image is contrasted but no corresponding voxel along its projection ray is estimated as contrasted. This can be due to the following reasons as shown in Figure 5: (a) the projection ray from a contrasted pixel does not intersect the left atrium as the LA has not been placed at the proper position yet; (b) the projection ray hits the LA, but all voxels intersected by this ray cannot contain CA because their corresponding pixels in the other plane are uncontrasted. Additional inconsistencies are introduced by pixels which are erroneously labeled as contrasted, for example, due to motion artifacts. To verify the validity of the CADE, we compute binary 2D images **C**_*T*_^A^, **C**_*T*_^B^ by forward projecting all contrasted voxels in *C*_*T*_^3D^ using *P*_A_, *P*_B_. We assess the consistency of the CADE for the given transformation *T* by computing the similarity between the fluoroscopic images and the projected CADE by

(8) $$\begin{array}{c} \rho_{\text{CADE}}\left(\;I_{\text{thr}}^{A},I_{\text{thr}}^{B},T\;\right)=\rho_{n}\left(\;I_{\text{thr}}^{A},C_{T}^{A}\;\right)\cdot \rho_{n}\left(\;I_{\text{thr}}^{B},C_{T}^{B}\;\right). \end{array}$$

**Figure 6:**
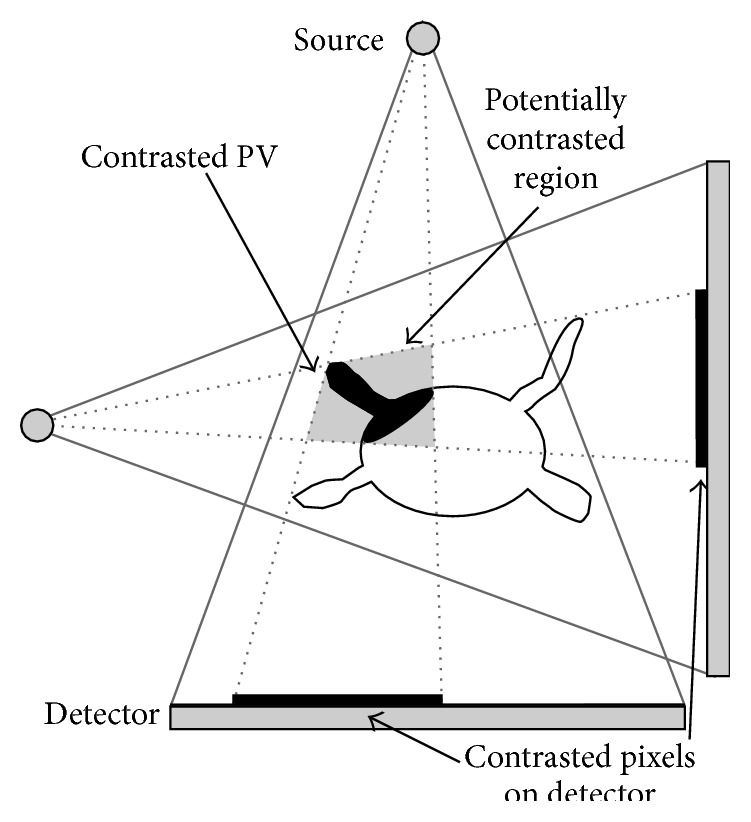
Top-down view on a left atrium with a contrasted pulmonary vein. The gray region denotes the area which may contain contrast agent based on the contrast agent observed in the 2D images. If the number of contrasted voxels in the volume was to be optimized, the optimization process would find a transformation such that as much of the potentially contrasted region as possible was included within the LA causing a registration bias towards larger LA regions.

Alternatively, the number of contrasted voxels inside the volume could be maximized. The set of potentially contrasted voxels is defined by the intersection of the projection ray bundles of all contrasted pixels from views A and B, respectively. Maximizing the number of contrasted voxels would lead to a transformation such that as much as possible of this set is contained inside the LA. If, for example, a pulmonary vein was filled with CA, the transformation would be chosen such that the set of possible contrasted pixels was located in the main body of the LA which provides the most space to include most possibly contrasted voxels; see Figure 6. To avoid this registration bias towards large structures inside the LA, we decided to optimize the consistency to the 2D images.

### 2.4. Confidence-Based Temporal Markov Filtering

To exploit dependencies between successive frames of a sequence, we model the position of the LA model in 3D as a time-dependent continuous Markov chain of the first order as proposed by us previously [22]. The states are transformations, that is, a translation and a rotation of the model. The parameter *τ*_*i*_ denotes the actual transformation of the LA in the *i*th contrasted frame, *T*_*i*_ refers to the estimate of the transformation for the *i*th frame, and *P*(*τ*_*i*_ = *T*_*i*_) is the probability that for frame *i* the transformation *T*_*i*_ is observed. For convenience, we define *P*(*T*_*i*_) = *P*(*τ*_*i*_ = *T*_*i*_). The transition probability from one state into another is independent of the frame number *i*. It is denoted as *P*(*T*_*i*_ → *T*_*i*+1_). Finally, a sequence of transformations *T*_1_,…, *T*_*n*_ is to be determined such that the term

(9) $$\begin{array}{c} P\left(\;T_{1},\ldots ,T_{n}\;\right)=P\left(\;T_{1}\;\right)\cdot \overset{n}{\underset{t=2}{\prod }}\left(\;P\left(\;T_{t-1}⟶T_{t}\;\right)\cdot P\left(\;T_{t}\;\right)\;\right) \end{array}$$

is maximized.

Transition probabilities and state probabilities control the filtering differently: Due to the transition probabilities, small motions of the LA from a frame to its next frame are preferred. This results in an averaging of the LA transformation over time. The state probability determines the impact of the averaging, that is, how much the registration result is changed by the filtering. We will model the state probability based on a confidence measure such that frames with high-confidence registration results are less subject to temporal filtering. If the confidence value is low, that is, the estimated error of the registration result is higher, we want to rely more on the results of the previous and next frame. Thus, high temporal averaging is performed for frames with a low confidence value. The state probabilities and the transition probability are determined as follows.

#### 2.4.1. State Probability

The contrast agent visible in frame *i* determines the probability of the LA for being transformed by *T*_*i*_ in this frame. If the dependency on other frames is ignored, the most probable transformation *T*_*i*_′ is the transformation obtained by optimizing the similarity measures *ρ* defined in (3), (5), and (8) or a combination of them.

The similarity measure *ρ*(**I** ^A,*i*^, **I** ^B,*i*^, *T*_*i*_′) depends on the contents of images **I** ^A,*i*^, **I** ^B,*i*^. Zhao et al. suggested using the value *ρ*(**I** ^A,*i*^, **I** ^B,*i*^, *T*_*i*_′) as a confidence measure: from all *N* registration results the frame *i* where *ρ*(**I** ^A,*i*^, **I** ^B,*i*^, *T*_*i*_′) is maximum should be selected as registration result for the complete sequence. We confirmed a correlation of *ρ* and the registration error. The value of *ρ* can therefore be used as a confidence measure. By linear regression, a function *e*(*ρ*(**I** ^A,*i*^, **I** ^B,*i*^, *T*_*i*_′)) can be determined to estimate the error based on the value of *ρ*. With the transformation *T*_*i*_′ of the LA found during optimization for frame *i*, the probability *P*(*T*_*i*_) can be modelled as normal distribution

(10) $$\begin{array}{c} P\left(\;T_{i}\;\right)=N\left(\;T_{i};T_{i}^{\prime },\Sigma_{i}\;\right). \end{array}$$

The confidence range is incorporated by setting the covariance matrix Σ_*i*_ to 1 · *e*(*ρ*(**I** ^A,*i*^, **I** ^B,*i*^, *T*_*i*_′)).

#### 2.4.2. Transition Probability

The transition probability *P*(*T*_*t*−1_ → *T*_*t*_) states how likely a movement of the LA is from frame *t* − 1 to *t*. Over multiple breathing cycles, the LA moves about a mean position. So the mean transformation is the identity. The likelihood of a transformation change depends on the magnitude of the change. The larger the change in translation and rotation is, the less likely the transformation transition is. To account for different frame rates, we consider the translational and rotational velocity **v** ∈ *ℝ* ^6^ which comprises three translational velocities and three rotational velocities. In a training step, velocity vectors are computed for annotated data. The covariance matrix Σ_**v**_ of the velocities gives an estimate for how likely a transition from one transformation to the next is. We model the probability of a transition *T*_*t*−1_ → *T*_*t*_ as a normal distribution

(11) $$\begin{array}{c} P\left(\;T_{t-1}⟶T_{t}\;\right)=N\left(\;\left(\;T_{t}-T_{t-1}\;\right)\cdot r;0,\Sigma_{v}\;\right). \end{array}$$

Besides the frame rate *r*, the transition probability depends also on the current breathing phase. Compared to breathing motion, cardiac motion can be neglected as it rather deforms the LA. If information on the breathing phase can be estimated [24], superior motion should be, for example, more likely during the inhale phase. During exhalation phase, inferior motion should have a higher probability. To account for breathing motion, for example, the mean value and the covariance matrix could be estimated separately for each stage of the breathing cycle.

#### 2.4.3. Most Probable State Sequence

The most likely state sequence,

(12) $$\begin{array}{c} T_{1}^{*},\ldots ,T_{n}^{*}=\underset{T_{i},\ldots ,T_{n}}{\arg\;\max}\;P\left(\;T_{i},\ldots ,T_{n}\;\right), \end{array}$$

can be found using a log-likelihood method: By applying the logarithm to (9), we get

(13) T1∗,…,Tn∗=arg maxT1⋯Tn⁡−12T1−T1′TΣ1−1T1−T1′+∑i=2nTi−Ti′TΣi−1Ti−Ti′+r·Ti−Txi−1TΣv−1Ti−Ti−1·r.

This convex optimization problem can be solved using the BFGS method.

## 3. Experiments and Results

We retrospectively evaluated our method on 21 clinical biplane X-ray sequences from 10 different patients. All of the patients provided their informed consent for the analysis of their clinical data. For all patients, a segmentation of their left atrium from a preoperatively acquired MRI scan was available as a triangle mesh. When using the CADE measure, this mesh was converted into a binary volume. The data set comprised 11 sequences showing an initial contrast agent injection where 15 mL CA was injected into the LA center through a sheath to verify the success of the transseptal puncture. Besides the sheath, only a coronary sinus catheter was present. There were 10 more sequences showing subsequent injections to verify catheter placement. In these cases, about 10 mL CA was injected and additional catheters were present. All X-ray angiography sequences were acquired during normal breathing using a standard acquisition protocol. This resulted in a total number of 133 contrasted frames. For our experiments, the first contrasted frame was determined manually. For 129 frames, reference registrations performed by three clinical experts were available; for the remaining four frames registrations performed by two clinical experts were available. Reference 3D registrations were established by manually shifting the mesh in each of two orthogonal views. The resulting 3D translation was refined this way until a good match to the associated contrast distributions as seen in the two 2D X-ray projections had been found. These reference registrations covered only 3D translation for the following reasons: First, patients were positioned head first, supine, during both preoperative and intraoperative imaging. This patient positioning rules out large degrees of rotation a priori. Furthermore, given the small amounts of contrast injected in our cases, the remaining small rotations were very difficult to detect. This made it practically impossible for our clinical experts to reliably correct them. This is why we decided to evaluate our approach without rotation.

As initialization for optimization, the 3D model was placed at that 3D position which corresponded to the centers of both 2D images. In some cases, the initialization was more than 30 mm away from the correct solution and beyond the capture range for gradient-based methods. Therefore we applied an octree-like coarse-to-fine scheme where we evaluated several positions at a coarse resolution. At positions in space that yielded a good similarity value, we performed subsequent evaluations at an increasingly finer resolution. The 3D translation $\hat{t}=arg\;max_{t}\rho (t)$ found by the optimization process of the respective objective function *ρ* was compared to the mean translation vector **t** ^*∗*^ of the three manual registration results. The distance ${\left\|\hat{t}-t^{*}\right\|}_{2}$ was used as error measure. The significance of the results was measured using a Wilcoxon signed-rank test [25] and a significance level of *p* = 0.05.

All sequences contained 12-bit images of size 1024 × 1024 pixels; all image processing, including rendering from the 3D model, was performed on the full image size. The kernel size for median filter was 30 pixels; the value *s* of (4) was 0.1. The standard deviation of the DOG filter was 24 pixels.

In the evaluation, we compared the similarity measures *ρ*_shad_^thr^, *ρ*_shad_^DSA^, *ρ*_CADE_, and *ρ*_edge_ and the combined similarity measures *ρ*_shad_^thr^ + *αρ*_edge_, *ρ*_shad_^DSA^ + *αρ*_edge_, and *ρ*_CADE_ + *αρ*_edge_. We investigated different weightings. Giving both terms equal weights, that is, setting *α* = 1, turned out to be a good choice. We did two types of evaluation: an evaluation on all frames and an evaluation using only a single frame of each sequence, namely, the one which provides the best similarity measure.

### 3.1. Evaluation on All Frames

We computed a registration for each contrasted frame and compared the result to the manual registration of the physicians. These results are clinically relevant if the physician requires a registration for a desired frame of his choice, for example, depending on the breathing phase. As potentially every frame could be selected by the physician, the overall registration accuracy should be high. The overall accuracy is also of importance if the results are postprocessed by temporal filtering.

The evaluation results are presented in Table 1. The overall interuser variability observed in the manual registrations was 3.2 ± 2.3 mm. Considering all sequences, *ρ*_CADE_ performed significantly better than *ρ*_shad_^thr^, *ρ*_shad_^DSA^, and the state-of-the-art method by Thivierge-Gaulin et al. [16]. Although *ρ*_edge_ gave significantly worse results than all other measures, a combination with *ρ*_edge_ could improve the results of *ρ*_shad_^thr^ and *ρ*_shad_^DSA^ significantly. For subsequent injections, *ρ*_CADE_ + *ρ*_edge_ performed significantly better than *ρ*_shad_^thr^, *ρ*_shad_^DSA^, their respective combinations with *ρ*_edge_, and the method by Thivierge-Gaulin et al. [16].

**Figure 7:**
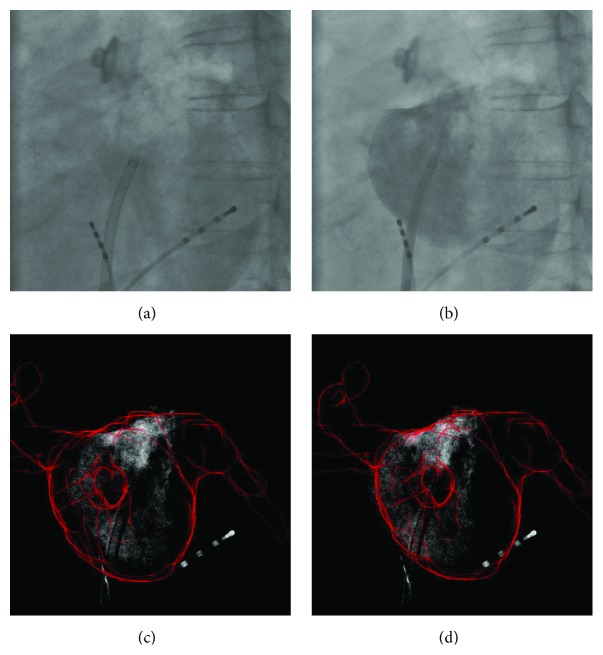
Uncontrasted (a) and contrasted (b) frame of an X-ray angiography. The registration result when using *ρ*_shad_^DSA^ (c) had an error of 6.7 mm. By using *ρ*_CADE_ + *ρ*_edge_ (d), the left border of the LA model fits better to the left edge of the CA and the error reduced to 3.1 mm.

**Figure 8:**
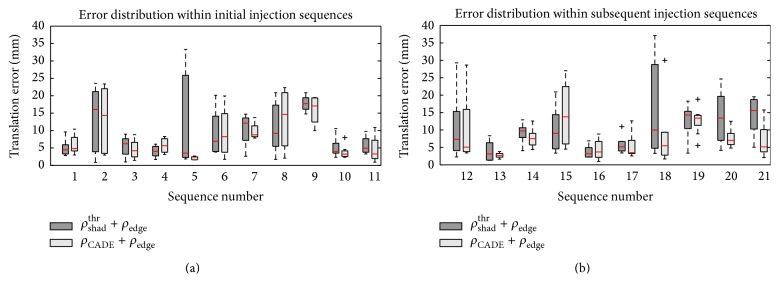
Distribution of the errors within each sequence. While there is no significant difference for initial injections (a), *ρ*_CADE_ + *ρ*_edge_ performs significantly better than *ρ*_shad_^thr^ + *ρ*_edge_ for subsequent injections (b). Subsequent contrast injections are characterized by a smaller amount of contrast and the presence of more catheters making registration more difficult. Example images of sequence 1 are shown in Figures 2 and 3; example images of sequence 2 are shown in Figure 1(a). Here, contrast agent is very faint and in some frames contrasted is already ejected into the left ventricle. Sequence 5 is shown in Figure 1(d). In this case, *ρ*_shad_^thr^ + *ρ*_edge_ got confused by edges caused by motion artifacts.

**Figure 9:**
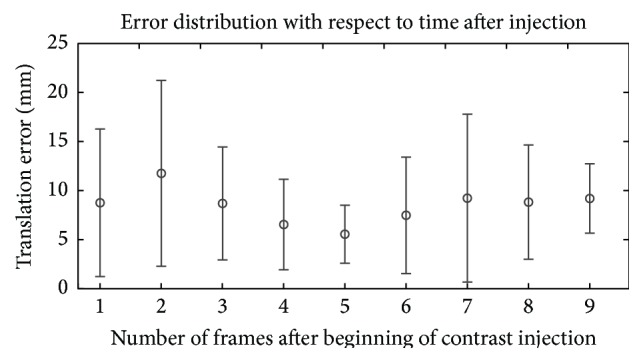
Average registration errors of *ρ*_CADE_ + *ρ*_edge_ depending on the position of the frame after start of the contrast agent injection calculated over all sequences. Frame 1 denotes the first frame which contained contrast agent. The average error at the second frame after the CA injection was 11.8 mm ± 9.5 mm; the average error at the fifth frame was 5.5 mm ± 3.0 mm. The frame rate of all sequences was 7.5 fps. Assuming a heart rate of 60 bpm, one heart cycle takes approximately 7 frames. So a first portion of CA is ejected into the ventricle not later than at the 7th frame. Note that the number of sequences used for calculation was not constant for each frame number as several sequences had fewer than nine contrasted frames.

For *ρ*_shad_^thr^ + *ρ*_edge_ and *ρ*_CADE_ + *ρ*_edge_, the error distribution for each sequence is shown in Figure 8, first for all initial injections and then for subsequent injections. In Figure 9, the error distribution is given for each frame number as counted after the CA injection. An example for a result is shown in Figure 7.

### 3.2. Evaluation on a Single Frame

Figure 8 indicates that many sequences have at least one frame with a registration error of less than 5 mm. In fact, there exists such a frame for over 75% of all sequences when using *ρ*_shad_^DSA^ or *ρ*_shad_^thr^ and for over 85% of the sequences when using *ρ*_CADE_, *ρ*_shad_^DSA^ + *ρ*_edge_, *ρ*_shad_^thr^ + *ρ*_edge_, or *ρ*_CADE_ + *ρ*_edge_. Zhao et al. [17] proposed to automatically find a good frame in each sequence and consider only these single good frames for evaluation. In other words, from all frames of a sequence, a single frame was automatically chosen for registration. From a clinical point of view, this would, however, only make sense if the physician accepted an automatically selected frame for registration or if further motion compensation steps for the other frames were done, for example, using device tracking [26].

Zhao et al. suggested selecting this single frame as follows: For each frame *i* ∈ [1, *m*] out of the *m* contrasted frames, the transform **t**_*i*_ that maximizes the similarity measure *ρ* for this frame is computed. In a second step, going through all frames of a sequence, the frame with the highest overall similarity measure is picked. The associated transform is denoted by ${\hat{t}}_{i}$. The error to the average manual registration result **t**_*i*_^*∗*^ is then computed as

(14) t^i−ti∗2with  t^i=arg maxi⁡ ρIA,i,IB,i,ti,  ti=arg maxt⁡ ρIA,i,IB,i,t.

The evaluation results are presented in Table 2. Recalling Section 3.1, where we found that the CADE method yielded good results for all frames, the CADE performance for a single frame was, however, not that good. This is why we investigated if a different single-frame selection strategy would lead to better results. Instead of using *ρ*_CADE_ both for translation estimation and for best-frame selection, we used *ρ*_CADE_ only for estimating the translation **t**_*i*_ for each frame *i*. Among the obtained translations **t**_*i*_, we selected the frame *i* with the corresponding translation ${\hat{t}}_{i}$ such that *ρ*_shad_^thr^ was maximized. So (14) was modified to

(15) $$\begin{array}{c} {\left\|\;{\hat{t}}_{i}-t_{i}^{*}\;\right\|}_{2}\;\text{with }{\hat{t}}_{i}=\underset{i}{\arg\;\max}\;\rho_{\text{shad}}^{\text{thr}}\left(I_{\text{thr}}^{A,i},I_{\text{thr}}^{B,i},t_{i}\right),\;\;t_{i}=\underset{t}{\arg\;\max}\;\rho_{\text{CADE}}\left(I_{\text{thr}}^{A,i},I_{\text{thr}}^{B,i},t\right). \end{array}$$

The results of this frame selection method are denoted by *ρ*_CADE_⊳*ρ*_shad_^thr^. We did the same also for the combination with *ρ*_edge_. Although only 10 sequences were available for initial contrast injections, *ρ*_shad_^DSA^, *ρ*_shad_^thr^ + *ρ*_edge_, and (*ρ*_CADE_ + *ρ*_edge_)⊳(*ρ*_shad_^thr^ + *ρ*_edge_) gave significant better results when compared to *ρ*_shad_^thr^. Also the performance of *ρ*_edge_ was significantly less. For subsequent injections, our proposed method *ρ*_CADE_⊳*ρ*_shad_^thr^ and the corresponding combination with *ρ*_edge_ outperformed the method by Thivierge-Gaulin et al.

### 3.3. Temporal Filtering

The error estimate function *e*(*ρ*(·)) and the transition probability covariance matrix Σ_**v**_ were trained in a leave-one-patient-out cross-validation. The results for the temporal filtered frames are given in Table 3. For a combination of the CADE and edge similarity measure, Markov filtering reduced the mean error to 5.7 ± 4.6 mm for initial injections and 6.6 ± 3.6 mm for subsequent injections. The respective median errors were 4.0 mm and 5.7 mm. The results obtained by the Markov filtering were in all cases significantly better than the results without filtering.

### 3.4. Runtime Performance

The image preprocessing took 0.5 s on an Intel Xeon E3 with 3.4 GHz and 16 GB RAM. The evaluations of the similarity measures were performed completely on the GPU. On an NVIDIA GeForce GTX 660 the evaluation of *ρ*_shad_^DSA^, *ρ*_shad_^thr^, or *ρ*_edge_ took 1.8 ms for a given translation and 13.4 ± 3.7 ms for *ρ*_CADE_. The whole registration for a single frame took 2.9 s for *ρ*_shad_^DSA^ or *ρ*_shad_^thr^ and 21.5 ± 5.9 s for *ρ*_CADE_. For a combination with *ρ*_edge_ it took 5.8 s and 27.1 ± 5.9 s, respectively. The runtime of the Markov filtering was 173 ± 124 ms per sequence.

## 4. Discussion and Conclusions

### 4.1. Similarity Measures

**Figure 10:**
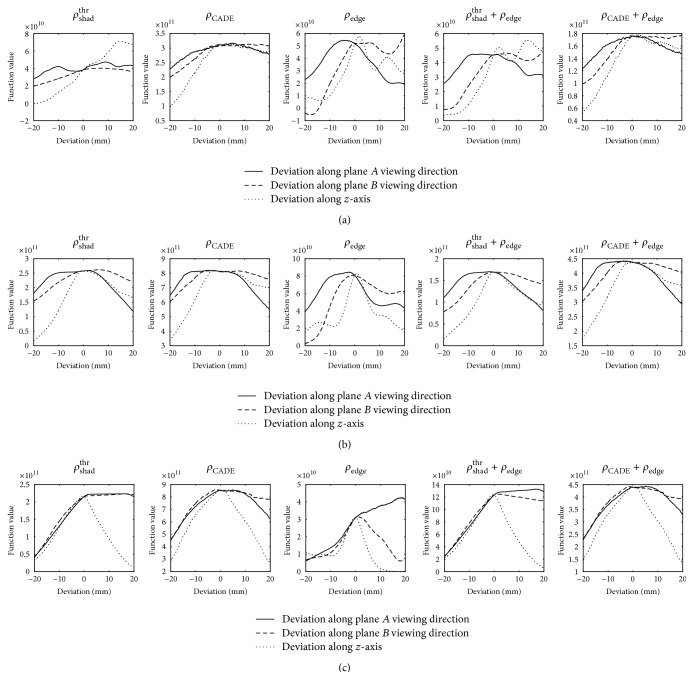
Optimization function values along sections through the ground-truth position. The sections were done along the viewing direction of the detector in plane A, the corresponding orthogonal direction in the transverse plane, and the *z*-axis of the patient coordinate system which corresponds to the cranial-caudal axis. The intersections are shown for three different frames: (a) one of the first frames of an initial injection (see Figure 3), (b) a frame of the same injection but with large parts of the left atrium contrasted, and (c) a secondary injection into a pulmonary vein (see Figure 1(g)). The plots for *ρ*_shad_^DSA^ were left out as they look very similar to those for *ρ*_shad_^thr^.

Our novel CADE-based method outperformed the shadow based similarity measures *ρ*_shad_^DSA^ and *ρ*_shad_^thr^, especially for reregistration sequences where only a small amount of contrast agent was used. The problem with nondistinct optima for shadow based similarity measures which was already sketched in Figure 2 becomes also apparent in the sections of the optimization function in Figure 10 for *ρ*_shad_^thr^: for a small amount of contrast agent like in Figure 10(a), the optimum is not at the position of the ground truth, but about 15 mm off. Also for secondary injections into small structures such as the pulmonary veins, the objective function for *ρ*_shad_^thr^ in Figure 10(c) has a large plateau. In both cases, *ρ*_CADE_ is more distinct, although clear extrema as for bones [12] are not obtained. Especially for secondary injections where CA was often injected into pulmonary veins, registration errors leading to inconsistent results could be avoided by *ρ*_CADE_. For well-contrasted sequences, the improvement by *ρ*_CADE_ was less as inconsistencies played only a minor role and the shape of the contrasted region in the image was more similar to the projected shadow of the left atrium. This becomes also apparent in Figure 10(b) where the shapes of the objective function of *ρ*_shad_^thr^ and *ρ*_CADE_ look very similar.

We found that the similarity measure using explicit apparent edges, *ρ*_edge_, yielded poor results when used on its own. A possible reason is that the objective function of *ρ*_edge_ has several local optima and also the global optimum does not necessarily correspond to the ground-truth position. However, *ρ*_edge_ can improve results significantly when combined with other similarity measures. It often has a more distinct local optimum at the position of the ground truth that facilitates fine registration. This is important as the other similarity measures define a rather plateau-shaped optimal region.

### 4.2. Time-Dependency

Figure 9 shows that the accuracy of the registration depends on when the frame was recorded after contrast agent injection. The best results were achieved at the 5th frame. This was in many cases the frame before the ejection into the ventricle; that is, it contains the most contrast agent. Especially for the first frames, where only little CA was present, registration accuracy was lower. The first frame is an exception, as it has a lower mean error than the second frame. This is probably because the previous frame, that is, the last uncontrasted frame, is used as mask frame for DSA computation which leads to low motion artifacts and a better extraction of the contrasted region.

### 4.3. Best-Frame Selection

We found that, for our registration to work best, a well opacified frame should be selected from the sequence. This strategy was evaluated in the context of the single-frame evaluation by selecting the frame which had the best objective function value. For methods like *ρ*_shad_^DSA^ and *ρ*_shad_^thr^, which are based on the projection shadow, the best frame usually corresponded to the most contrasted frame. The measure *ρ*_CADE_, however, is not related to the amount of contrast agent, but it depends on consistency. When relying on *ρ*_CADE_ for best-frame selection, we get the frame yielding the most consistent registration. This is, however, not necessarily the frame with the most contrast agent. And it is usually the frame with the most contrast agent which provides the most information for registration. We believe that this is the reason why the errors in Table 2 for *ρ*_CADE_ are higher. If, however, from the translations estimated by *ρ*_CADE_ + *ρ*_edge_, the single frame was selected based on *ρ*_shad_^thr^ + *ρ*_edge_, better results were achieved as now again the frames with the most contrast agent were selected.

Sometimes, the automatic best-frame selection does not provide a frame with a satisfactory result. Still, 85% of all sequences contain at least one frame that is below a clinical relevant threshold of 5 mm [27, 28]. To benefit from the fact that at least one frame with a good registration result is likely to be found, the frame selection could be performed manually by stepping through the frames and the associated registration results. The user can then quickly select the frame with the best registration result. Although this implementation still involves user input, the required user interaction is less than for fully manual registration.

For the remaining 15% of the cases or if higher accuracy level, for example, 3 mm [29], is required, small manual adjustment may be necessary in these cases.

### 4.4. Temporal Filtering

Temporal filtering based on the Markov chain could reduce the error significantly. Particularly high errors for frames at the beginning or at the end of the contrast injection were reduced. If a registration is not desired for an arbitrary frame but rather for a frame determined by the physician, for example, based on the breathing phase, temporal filtering should be applied.

### 4.5. Runtime Performance

It is notable that the runtime for *ρ*_CADE_ was considerably higher compared to, for example, *ρ*_shad_^thr^. This is because the contrast agent distribution needs to be updated in each iteration before projecting it. Moreover, this part of the implementation has not yet been optimized. Although the fast runtime of, for example, *ρ*_shad_^thr^ will remain out of reach, we are confident that the runtime for *ρ*_CADE_ could be reduced to a clinically acceptable level.

### 4.6. Impact of Clinical Issues

In general, the performance for initial registration was better, as the amount of contrast agent was higher and fewer catheter artifacts were present. We found by visual inspection that also the amount of breathing motion had an impact on the registration accuracy as it caused motion artifacts in the DSA computation. As a consequence, if the patient was not anesthetized, acquisition of the CA injection should be performed under breath-hold or shallow breathing. For cases with intubation, also jet ventilation [30] or a small period of apnea could be applied to reduce breathing motion. Since our data was not acquired using a dedicated DSA program, the different brightness levels within a sequence changed. This interfered with the subtraction image computation. Although intuitively appealing, our data did not allow us to make a statement if the registration accuracy depends on the part of the LA in which CA was injected.

For initial contrast injections, an average accuracy of 4.6 ± 4.0 mm and a median error of 3.7 mm could be achieved using *ρ*_CADE_ + *ρ*_edge_ together with the best-frame selection approach. This error is in the range of the interuser variability of 3.2 ± 2.3 mm. The faster registration method using *ρ*_shad_^thr^ + *ρ*_edge_ reached an accuracy of 4.8 ± 4.6 mm. These numbers are also similar to the accuracy reported when performing manual registration based on segmentations of the CS, the whole heart, and the spine [7] or a segmentation of the CS when 3D and 2D data are in the same breathing and cardiac phase [31] which was achieved by ventilation and rapid pacing of anesthetized patients. However, our method does not require structures other than the LA to be segmented and requires no anesthesia. Compared to a registration based on the CS alone [32], the error was reduced by over 50%.

The average accuracy for all frames after applying temporal filtering is 5.7 mm for *ρ*_CADE_ + *ρ*_edge_ and 6.1 mm for *ρ*_shad_^thr^ + *ρ*_edge_. Though the results of *ρ*_CADE_ + *ρ*_edge_ are close to the 5 mm threshold [27, 28], it remains open if this is sufficient for a clinical application, but we believe that these results should at least provide users with an acceptable initial estimate for further manual adjustments.

### 4.7. Future Work

By now, the registration method was evaluated only for left atria. In a next step, the performance of this approach to contrast-based registration of the right atrium should be evaluated. A registration based on the right atrium would also provide a registration for the LA which is available for transseptal puncture or one of the ventricles could be assessed. Due to the lack of pulmonary veins and arteries, the ventricles have a more simple anatomical structure but are subject to stronger cardiac motion. How strongly this affects the registration accuracy is open.

## 5. Conclusions

Compared to the approach by Zhao et al. [17], the use of special weights for different heart regions is not needed for any of the proposed approaches. In addition, for *ρ*_shad_^thr^ + *ρ*_edge_, a time-consuming DRR generation can be avoided. As a result, a registration approach based on a combination of shadow and edge features can be computed fast. If sufficient computational power was available, the novel CADE-based measure, which takes consistency into account, should be used as it improves results significantly, especially when very small amounts of contrast agent are injected.

## Figures and Tables

**Table 1:** Translation errors for all frames.

Objective function	Initial injection	Subsequent injections
Thivierge-Gaulin	11.4 ± 8.8 mm	10.4 ± 5.4 mm
*ρ*_shad_^DSA^	9.3 ± 6.9 mm	9.8 ± 4.8 mm
*ρ*_shad_^thr^	9.9 ± 7.9 mm	12.1 ± 9.1 mm
*ρ*_edge_	12.0 ± 8.9 mm	14.6 ± 8.7 mm
*ρ*_CADE_	**8.7 ± 6.4 mm**	**9.0 ± 5.9 mm **

*ρ*_shad_^DSA^ + *ρ*_edge_	8.3 ± 6.6 mm	9.6 ± 5.4 mm
*ρ*_shad_^thr^ + *ρ*_edge_	8.3 ± 6.7 mm	10.5 ± 7.6 mm
*ρ*_CADE_ + *ρ*_edge_	**7.9 ± 6.3 mm**	**8.8 ± 6.7 mm**

Clinical experts	3.3 ± 2.7 mm	3.1 ± 1.7 mm

**Table 2:** Translation errors for a single automatically chosen frame as described in (14) and (15).

Objective function	Initial injection	Subsequent injections
Thivierge-Gaulin	8.3 ± 8.2 mm	10.6 ± 4.4 mm
*ρ*_shad_^DSA^	5.1 ± 3.8 mm	9.6 ± 5.1 mm
*ρ*_shad_^thr^	7.1 ± 4.3 mm	8.1 ± 6.6 mm
*ρ*_edge_	14.1 ± 10.0 mm	12.1 ± 9.1 mm
*ρ*_CADE_	6.0 ± 5.0 mm	**7.0 ± 3.8 mm**
*ρ*_CADE_⊳*ρ*_shad_^thr^	***5.0 ± 5.0 mm***	*7.7 ± 4.9 mm *

*ρ*_shad_^DSA^ + *ρ*_edge_	5.1 ± 4.2 mm	8.5 ± 4.7 mm
*ρ*_shad_^thr^ + *ρ*_edge_	4.8 ± 4.6 mm	7.8 ± 5.7 mm
*ρ*_CADE_ + *ρ*_edge_	6.1 ± 5.8 mm	7.6 ± 5.5 mm
(*ρ*_CADE_ + *ρ*_edge_)⊳ (*ρ*_shad_^thr^ + *ρ*_edge_)	***4.6 ± 4.0 mm***	***7.3 ± 5.2 mm***

**Table 3:** Translation errors for temporal filtered frames.

Objective function	Initial injection	Subsequent injections
Thivierge-Gaulin	9.5 ± 6.2 mm	7.2 ± 3.0 mm
*ρ*_shad_^DSA^	7.5 ± 4.1 mm	7.7 ± 3.5 mm
*ρ*_shad_^thr^	7.5 ± 4.8 mm	9.1 ± 5.6 mm
*ρ*_edge_	9.7 ± 7.1 mm	12.9 ± 7.6 mm
*ρ*_CADE_	6.1 ± 4.1 mm	**6.2 ± 3.1 mm**

*ρ*_shad_^DSA^ + *ρ*_edge_	6.5 ± 4.3 mm	7.4 ± 3.3 mm
*ρ*_shad_^thr^ + *ρ*_edge_	6.1 ± 4.4 mm	8.1 ± 3.7 mm
*ρ*_CADE_ + *ρ*_edge_	**5.7 ± 4.6 mm**	6.6 ± 3.6 mm
